# Enhanced metabolic adaptations following late dark phase wheel running in high-fat diet-fed mice

**DOI:** 10.1016/j.molmet.2025.102116

**Published:** 2025-02-22

**Authors:** Stephen P. Ashcroft, Amy M. Ehrlich, Krzysztof Burek, Logan A. Pendergrast, Caio Y. Yonamine, Jonas T. Treebak, Juleen R. Zierath

**Affiliations:** 1 Novo Nordisk Foundation Center for Basic Metabolic Research, Faculty of Health and Medical Sciences, University of Copenhagen, Copenhagen, Denmark; 2 Integrative Physiology Department of Molecular Medicine and Surgery, Karolinska Institutet, Stockholm, Sweden; 3 Integrative Physiology Department of Physiology and Pharmacology, Karolinska Institutet, Stockholm, Sweden

**Keywords:** Exercise, Metabolism, Circadian

## Abstract

Exercise interventions represent an effective strategy to prevent and treat metabolic diseases and the time-of-day-dependent effects of exercise on metabolic outcomes are becoming increasingly apparent. We aimed to study the influence of time-restricted wheel running on whole-body energy and glucose homeostasis. Male, 8-week-old, C57BL/6NTac mice were fed either a 60% high-fat diet (HFD) or a 10% low-fat diet (LFD) for 4 weeks. Following this, mice were given access to a running wheel between zeitgeber time (ZT) 12–16 (early dark phase) or ZT 20-0 (late dark phase). Sedentary mice had access to a permanently locked wheel. Mice were housed under these conditions in metabolic chambers for 4 weeks in which LFD and HFD conditions were maintained. Following the exercise intervention, body composition and glucose tolerance were assessed. Wheel running during either the early or late dark phase resulted in metabolic improvements such as attenuation in body weight gain, enhanced glucose tolerance and reduced ectopic lipid deposition. However, late dark phase exercise resulted in a greater reduction in body weight gain, as well as enhanced metabolic flexibility and insulin sensitivity. Our data suggest that late dark phase versus early dark phase exercise confers greater metabolic adaptations in HFD-fed mice.

## Highlights

• Exercise-timing promotes divergent metabolic adaptations in lean and obese mice.
• Late dark phase exercise resulted in a greater reduction in body weight gain.
• Late dark phase exercise promotes metabolic flexibility and insulin sensitivity.
• Enhanced *Pdk4* expression following late dark phase exercise.

## 1 Introduction

Circadian rhythms are endogenous biological oscillations with a 24-hour periodicity, which are directly under the control of the molecular core clock. This clock is located within the hypothalamic suprachiasmatic nucleus (SCN) and consists of a transcriptional-translational feedback loop, which is synchronized to external light cues. The DNA-binding transcription factors, circadian locomotor output cycles kaput (CLOCK) and neuronal PAS domain protein 2 (NPAS2) heterodimerize with basic helix-loop-helix ARNT-like 1 (BMAL1) and direct the transcriptional activation of core clock-controlled genes including their own repressors, *Period* and *Cryptochrome* [1]. The same clock machinery is present in peripheral tissues and is synchronized to the SCN; however, environmental factors known as *zeitgebers* can entertain tissue-specific clocks. For example, alterations in activity patterns and the feed-fasting cycle can alter the phase of tissue-specific clocks within skeletal muscle and liver [2,3].

The relative importance of the circadian clock to whole-body and tissue-specific metabolism has been elucidated across multiple levels. Observational studies suggest perturbations to diurnal rhythms in the form of night-shift work are associated with the development of metabolic diseases such as obesity and type 2 diabetes [[4], [5], [6]]. Furthermore, circadian misalignment via a rapid day–night shift disrupts glucose homeostasis and impairs peripheral insulin sensitivity [[7], [8], [9]]. The molecular core clock not only activates the transcriptional activity of its own repressors but also controls the expression of genes related to lipid storage, insulin sensitivity and glucose metabolism [10]. In addition, whole-body *Clock* or *Bmal1* knockout mice exhibit increased susceptibility to obesity and metabolic syndrome [11,12]. Similar metabolic impairments including insulin resistance, reduced glucose uptake and increased adiposity are observed in liver, skeletal muscle and adipose tissue-specific *Bmal1* knockout mice [[13], [14], [15], [16]]. Therefore, the core clock is intrinsically linked to biological endpoints associated with the development of metabolic diseases and potential avenues to entrain the core clock may offer therapeutic benefit.

Exercise represents an effective strategy to prevent and treat metabolic disease and has the potential to entrain both the central and peripheral clocks [3,17]. Furthermore, exercise training facilitates improvements in insulin sensitivity among individuals with obesity and type 2 diabetes [18,19]. However, increasing evidence suggests the effects of exercise are time-dependent [20]. In rodents, exercise capacity is time-of-day-dependent, with timed exercise bouts resulting in divergent transcriptional and metabolomic responses in a tissue-specific manner [[21], [22], [23]]. The activation of divergent signaling pathways following acute exercise may alter the adaptive phenotype following chronic time-dependent exercise. In response to chronic exercise interventions, mice undergoing voluntary wheel running (VWR) during the late dark phase, corresponding to the late active phase, consistently display a lower body weight in comparison to mice running during the early dark phase [[24], [25], [26]]. Current evidence suggests that the effects of exercise-timing are unrelated to the total amount of energy intake or expenditure, although a detailed analyses across the full training period has not been performed [24,26]. Therefore, we aimed to investigate the effects of time-restricted VWR on whole-body energy homeostasis, body composition, and glucose tolerance in high-fat diet-fed mice. In addition, we utilized metabolic caging with the aim of defining the metabolic and behavioral mechanisms that lead to divergent adaptations with time-of-day restricted VWR exercise.

## 2 Materials and methods

### 2.1 Animal experiments

**Figure 1:**
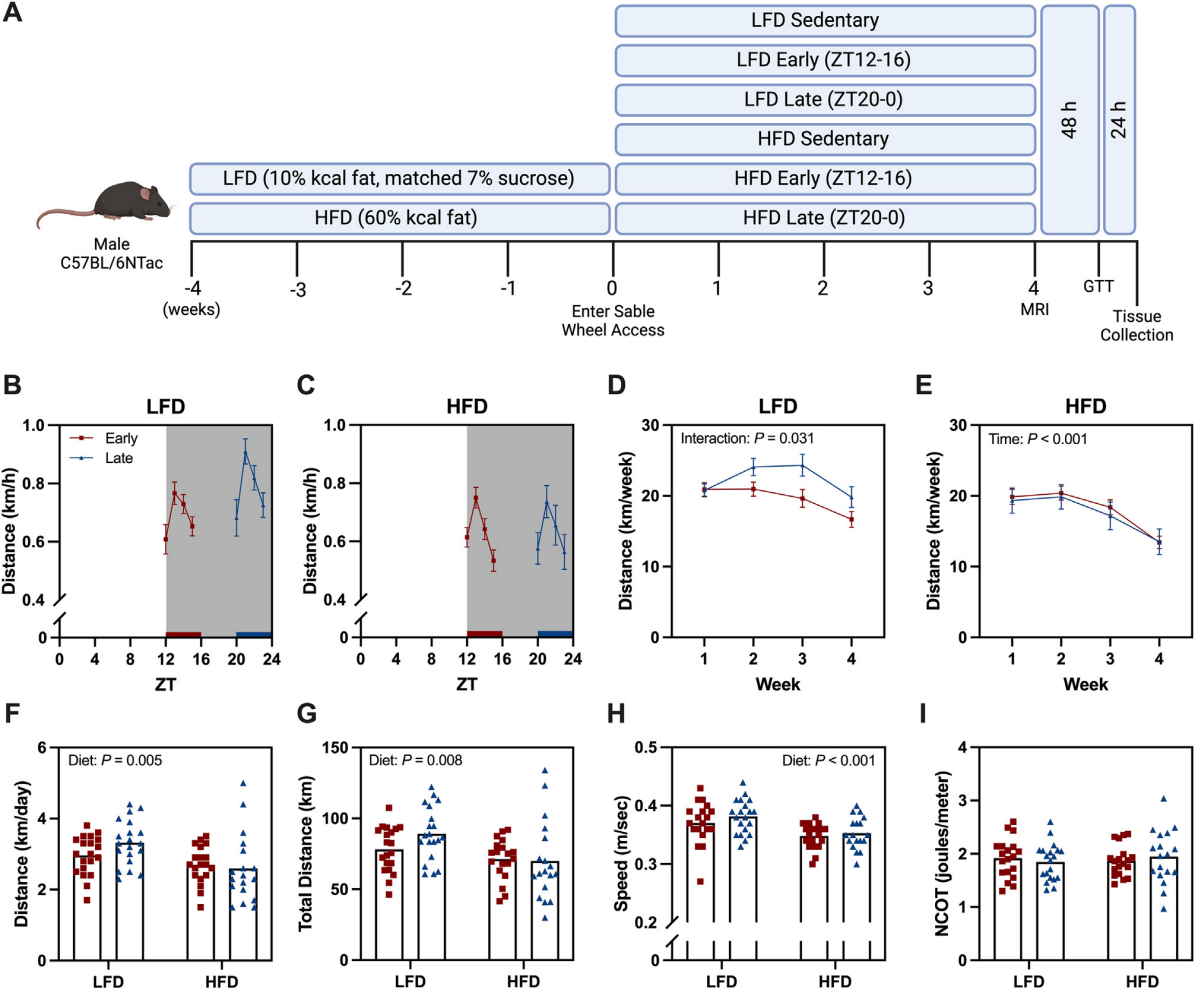
**Study protocol and wheel running behavior between early and late wheel access groups.** (A) Schematic of study overview. (B–C) Average running distance during a 24 h period in low-fat diet (LFD) and high-fat diet (HFD) mice. (D–E) Weekly total running distance in LFD and HFD mice. (F) Average daily distance across full training period. (G) Total distance across full training period. (H) Average speed during individual running bouts across full training period. (I) Net cost of transport (NCOT) during individual running bouts. Data are reported as mean ± SE or mean values with data points representing individual animals (n = 17–20/group).

Animal experiments were performed in compliance with the Directive 2010/63/EU of the European Parliament and were approved by the Danish Animal Experiments Inspectorate (license no. 2019-15-0201-01663). Male C57BL/6NTac mice were purchased from Taconic (Denmark) and arrived at 6 weeks of age. Mice were group-housed with *ad libitum* access to a standard chow (Altromin, #1310) and water under a 12:12 h light:dark cycle. Lights on was at 6 am and represents zeitgeber time (ZT) 0. At 8 weeks of age, mice were transitioned to either a 60% kcal high-fat diet (HFD, Research Diets, #D12492, 31.7% Lard & 3.2% Soybean Oil) or a 10% kcal low-fat diet (LFD, Research Diets, #D12450J, 1.9% Lard & 2.4% Soybean Oil) matched for sucrose content. Mice were maintained on respective LFD and HFD for a period of 4 weeks (Figure 1A).

### 2.2 Indirect calorimetry

Following 4 weeks of dietary intervention, mice were transferred to the Sable Systems Promethion Core. Mice were single-housed and given unrestricted access to a running wheel (11.4 cm diameter) for 48 h to promote wheel running behavior. Following this, mice were matched for body weight and divided into three groups per respective diet (Figure 1A). Mice were given wheel access during the first 4 h of the dark phase (ZT12-16) (early dark phase) or the final 4 h of the dark phase (ZT20-0) (late dark phase). The respective LFD and HFD sedentary control mice had access to a permanently locked wheel. Wheel running data from the whole period (0–4 weeks) were used to calculate daily and weekly average distance covered, total distance covered and the net cost of transport (NCOT). NCOT was calculated by regressing meters run per running bout against joules of net energy expended during each bout. Exclusion criteria were established prior to the study commencing and mice exhibiting poor running behavior (<1 km/day) were excluded (n = 6). Cumulative energy expenditure and intake was calculated during the whole period (0–4 weeks) whereas 24 h profile of respiratory exchange ratio (RER), energy intake, energy expenditure and non-wheel activity data were analyzed from a 5-day period in the 4th week of the intervention. Data were monitored in 15-minute periods and hourly averages were calculated to report diurnal patterns across 24 h. Daily amplitudes for RER, energy expenditure, energy intake and non-wheel activity levels were calculated by subtracting the minimum hourly value from the maximal hourly value [27].

### 2.3 Body composition

Body weight was measured weekly from 8 weeks of age. Following 4 weeks of wheel access, body composition was measured using the Minispec LF90 MRI scanner (Bruker).

### 2.4 Glucose tolerance test

Mice were housed without wheel access for 48 h to minimize the acute effects of exercise prior to the glucose tolerance test (GTT). The GTT was performed following a 4-h fast (ZT1 to ZT5). Glucose was delivered via oral gavage at a concentration of 2.5 g/kg of lean mass. Blood glucose levels were monitored from the tail-tip using a hand-held glucometer (Contour XT, Bayer) in the basal state and 15-, 30-, 60-, 90- and 120-minutes following glucose administration. Whole-blood insulin levels were determined in the basal state and 15-, 30-, 60- and 90-minutes following glucose administration using an insulin ELISA kit (Ultra-Sensitive Mouse Insulin ELISA Kit, Crystal Chem, #90080) as described [28]. Upon completion of the GTT, food access was returned.

### 2.5 Sample collection

Mice were sacrificed between the hours of ZT3-6 ∼24 h after the GTT. Mice were anesthetized via the intraperitoneal injection of 100 mg/kg of pentobarbital. Blood samples were collected in an EDTA coated tube and placed on ice. Samples were subjected to centrifugation at 2,000 *g* for 10 min at 4 °C and the resulting plasma supernatant was collected and stored at −80 °C. Following cervical dislocation, tissues were dissected and frozen in liquid nitrogen. Tissue samples were stored at −80 °C for later analysis.

### 2.6 Biochemical analyses

Plasma triglyceride concentrations were measured using the Triglyceride Assay (Randox, #TR210), while cholesterol levels were determined with the Cholesterol Assay (Randox, #CH200). All assays were conducted according with manufacturer's protocols. Samples were analyzed in duplicate utilizing 5 μL of plasma per measurement. Tissue triglyceride [27] and glycogen [29] contents were determined in liver and quadriceps (∼25 mg) following previously described methods.

### 2.7 RNA extraction

Powdered quadriceps (∼30 mg), liver (∼10 mg) and inguinal white adipose tissue (∼50 mg) were homogenized in Trizol (1 mL) with a steal bead using the Qiagen TissueLyser II (3 × 90 s at 30 Hz at 4 °C). Adipose tissue samples were subjected to centrifugation (16,000 *g* for 10 min at 4 °C) and the Trizol supernatant was pipetted from underneath the fat layer into a clean tube. Choloroform (200 μL) was added to each sample and samples were mixed for 30 s by shaking. Samples were subjected to centrifugation (14,000 *g* for 15 min at 4 °C). The aqueous phase (300 μL) was combined with an equivalent volume of 70% ethanol. RNA extraction was performed using the RNAeasy kit (Qiagen) according to the manufacturer's instructions with on-column DNA digestion. RNA quantification was performed with a spectrophotometer (Nanodrop 8000, Thermo Scientific).

### 2.8 cDNA synthesis and qPCR

cDNA synthesis was performed with 1 μg of RNA using the iScript cDNA synthesis kit (Bio-Rad). RT-qPCR was performed with SYBR Green (Primer Design) on a CFX384 Real-Time PCR system (Bio-Rad). Primers (Table 1) were used at a final concentration of 200 nM. Relative quantification was determined using the ΔΔ CT method and data were normalized to the housekeeping gene *18s*.

**Table 1:** Primer sequences for RT-qPCR analysis of genes involved in metabolism, circadian rhythms, and thermogenesis.

*Gene*	Forward (5′ to 3′)	Reverse (5′ to 3′)
*Glut4*	GTG ACT GGA ACA CTG GTC CTA	CCA GCC ACG TTG CAT TGT AG
*Hk2*	AGA GAA CAA GGG CGA GGA G	GGA AGC GGA CAT CAC AAT C
*Pdk4*	AGG GAG GTC GAG CTG TTC TC	GGA GTG TTC ACT AAG CGG TCA
*Cpt1b*	GTG CTG GAG GTG GCT TTG	TTT GCT GGA GAT GTG GAA GA
*Pgc1a*	CCC TGC CAT TGT TAA GAC C	TGC TGC TGT TCC TGT TTT C
*Pparg2*	GCA TGG TGC CTT CGC TGA	TGG CAT CTC TGT GTC AAC CAT G
*Ucp1*	CTG CCA GGA CAG TAC CCA AG	TCA GCT GTT CAA AGC ACA CA
*Cidea*	AAA GGG ACA GAA ATG GAC AC	TTG AGA CAG CCG AGG AAG
*Dio2*	AAG GCT GCC GAA TGT CAA CGA ATG	TGC TGG TTC AGA CTC ACC TTG GAA
*Prdm16*	CAG CAC GGT GAA GCC ATT C	GCG TGC ATC CGC TTG TG
*Elovl3*	TCG TCT ATC TGT TGC TCA TCG	CTG TTG CCA TAA ACT TCC ACA
*Arntl*	TAG GAT GTG ACC GAG GGA AG	TCA AAC AAG CTC TGG CCA AT
*Nr1d1*	GTC TCT CCG TTG GCA TGT CT	CCA AGT TCA TGG CGC TCT
*Npas2*	AAG GAT AGA GCA AAG AGA GCC T	CAT TTT CCG AGT GTT ACC AGG G
*Per1*	TGA AGC AAG ACC GGG AGA G	CAC ACA CGC CGT CAC ATC AA
*Per2*	AAT GGC CAA GAG GAG TCT CA	ATG CTT CCT TCT GTC CTC CA
*18s*	AGT CCC TGC CCT TTG TAC ACA	GAT CCG AGG GCC TCA CTA AAC

### 2.9 Statistical analyses

Data are presented as bars representing the mean, with individual values, which represent individual animals or means with error bars representing standard error (SE). A *p*-value of less than 0.05 was considered statistically significant. Two-way ANOVAs were used to test for a main effect of group, a main effect of diet, and a group × diet interaction. A Tukey's *post hoc* test was used to compare between groups if there was a main effect for group. A Sidak's *post hoc* test was performed if there was a group × diet interaction. ANOVAs were performed in GraphPad Prism (Version 10.1.1). For the associated GTT data, area under the curve (AUC) was calculated via the trapezoidal method. To test whether energy expenditure was different between wheel access groups, an ANCOVA was performed with energy expenditure as the dependent variable lean mass as a covariate, and wheel running group as a fixed factor. ANCOVA analysis was performed in SPSS (Version 29.0.2.0, IBM).

## 3 Results

### 3.1 Early and late wheel access mice display similar running behavior

During the respective 4-hour access periods, both early and late wheel access mice exhibited a similar running pattern regardless of diet (Figure 1B–C). In LFD- and HFD-fed mice, total running distance per week decreased over time (Figure 1D–E). HFD-fed mice ran shorter daily distances, covered less total distance, and maintained a lower average speed compared to LFD mice (Figure 1F–H). However, early and late wheel access mice show no differences in daily running distance, total running distance, or average speed (Figure 1F–H). Additionally, NCOT remained consistent across diets and groups, indicating that mice expend a similar amount of energy per distance run on the wheel (Figure 1I).

### 3.2 Greater attenuation of body weight gain following late dark phase exercise

**Figure 2:**
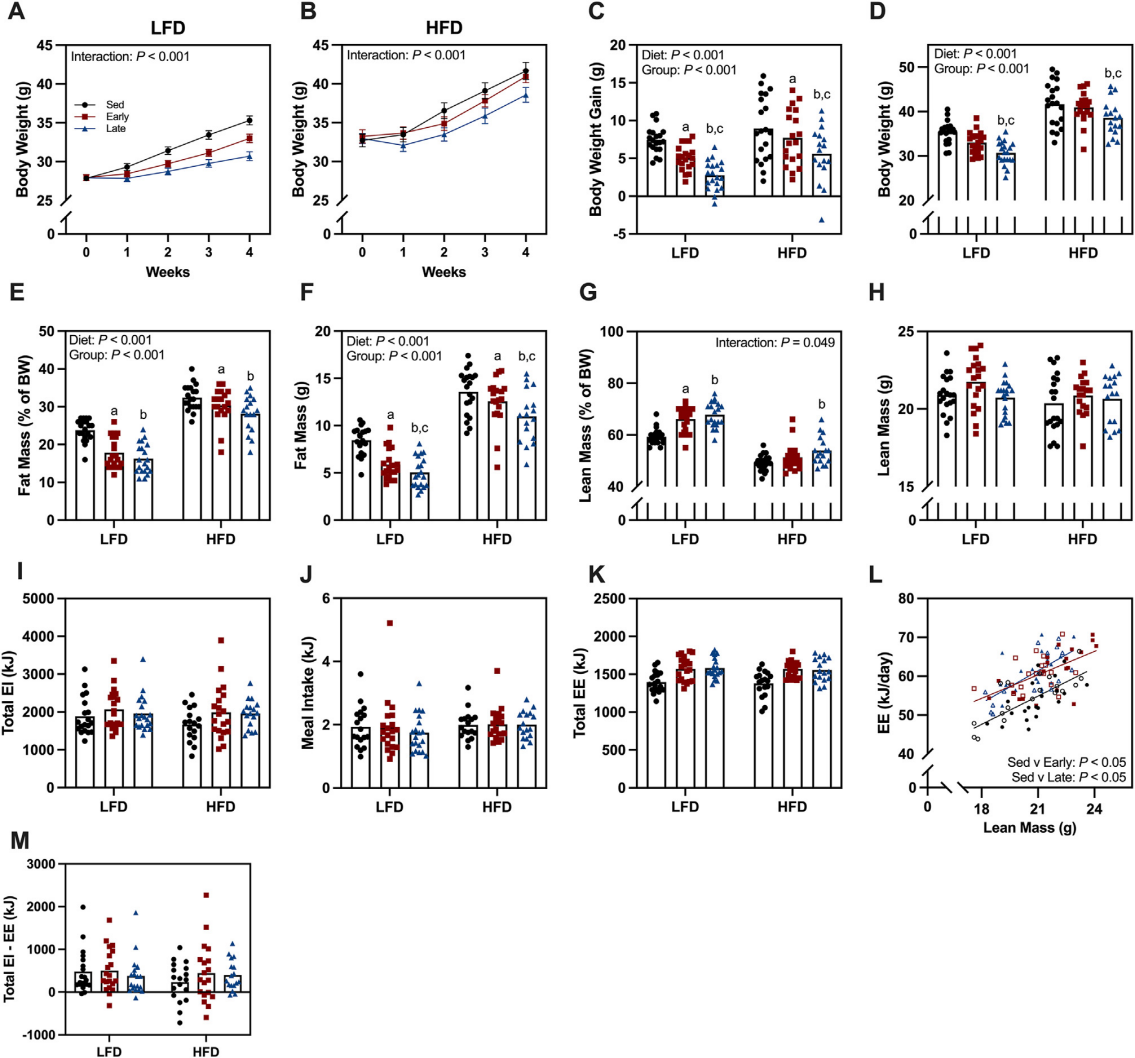
**Greater attenuation in body weight gain following late dark phase wheel running.** (A–B) Weekly body weight in low-fat diet (LFD) and high-fat diet (HFD) mice. (C) Body weight gain across the wheel running intervention. (D) Final body weight following wheel running intervention. (E) Fat mass as a percentage of body weight and (F) absolute fat mass following wheel running intervention. (G) Lean mass as a percentage of body weight and (H) absolute lean mass following wheel running intervention. (I) Cumulative energy intake (EI) during the full wheel running intervention. (J) Average EI during individual meal intake events. (K) Cumulative energy expenditure during the full wheel running intervention. (L) Energy expenditure as a function of absolute lean mass (closed symbols = LFD and open symbols = HFD). (M) Calculation of energy balance by subtracting total energy expenditure from total energy intake. Data are reported as mean ± SE or mean values with individual data points representing individual animals (n = 17–20/group). Statistical analysis was performed using a two-way ANOVA. ^a^Sed v Early, ^b^Sed v Late and ^c^Early v Late (*P* < 0.05). (K–L) Analyzed via ANCOVA.

Prior to the wheel-running intervention, sedentary, and early or late wheel running groups were matched for body weight (Figure 2A–B). Both LFD and HFD mice gained weight over the 4-week wheel-running intervention, but the rate of weight gain differed between wheel access groups (Figure 2A–B). As expected, HFD mice gained more weight and had a higher final body weight than LFD mice (Figure 2C–D). The exercise intervention reduced body weight gain in both early and late wheel access mice compared to respective sedentary controls (Figure 2C). However, body weight gain was further attenuated in late wheel access mice compared to the early wheel access mice (Figure 2C). This reduction in body weight gain led to a lower final body weight in late access mice compared to both sedentary and early wheel access mice (Figure 2D).

Following the wheel-running intervention, body composition was assessed via MRI. HFD mice had a higher absolute fat mass and fat mass percentage of body weight compared to LFD mice (Figure 2E–F). The exercise intervention lowered both absolute fat mass and fat mass percentage in early and late wheel access mice compared to sedentary mice (Figure 2E–F). Furthermore, late wheel access mice had a lower absolute fat mass than early wheel access mice (Figure 2F). Lean mass percentage varied depending on group and diet (Figure 2G). In LFD mice, both early and late wheel access groups had a higher lean mass as a percentage of body weight than sedentary mice (Figure 2G). However, in HFD mice, only the late wheel access group showed a higher lean mass percentage of body weight compared to sedentary mice (Figure 2G). Absolute lean mass did not differ across diet groups or wheel-running intervention (Figure 2H). We found no group- or diet-dependent differences in cumulative energy intake or the average feeding event (Figure 2I–J). In terms of energy expenditure, both early and late wheel access mice expended more energy than sedentary mice when adjusted for lean mass (Figure 2K-L). However, energy expenditure did not differ between early and late wheel access mice when lean mass was used as a covariate (Figure 2L). Finally, energy balance was calculated by subtracting total energy expenditure from the total energy intake. We found that most mice remained in a positive energy balance, consistent with weight gain throughout the intervention, with no differences between groups or diets (Figure 2M).

### 3.3 Late dark phase wheel running alters diurnal substrate metabolism

**Figure 3:**
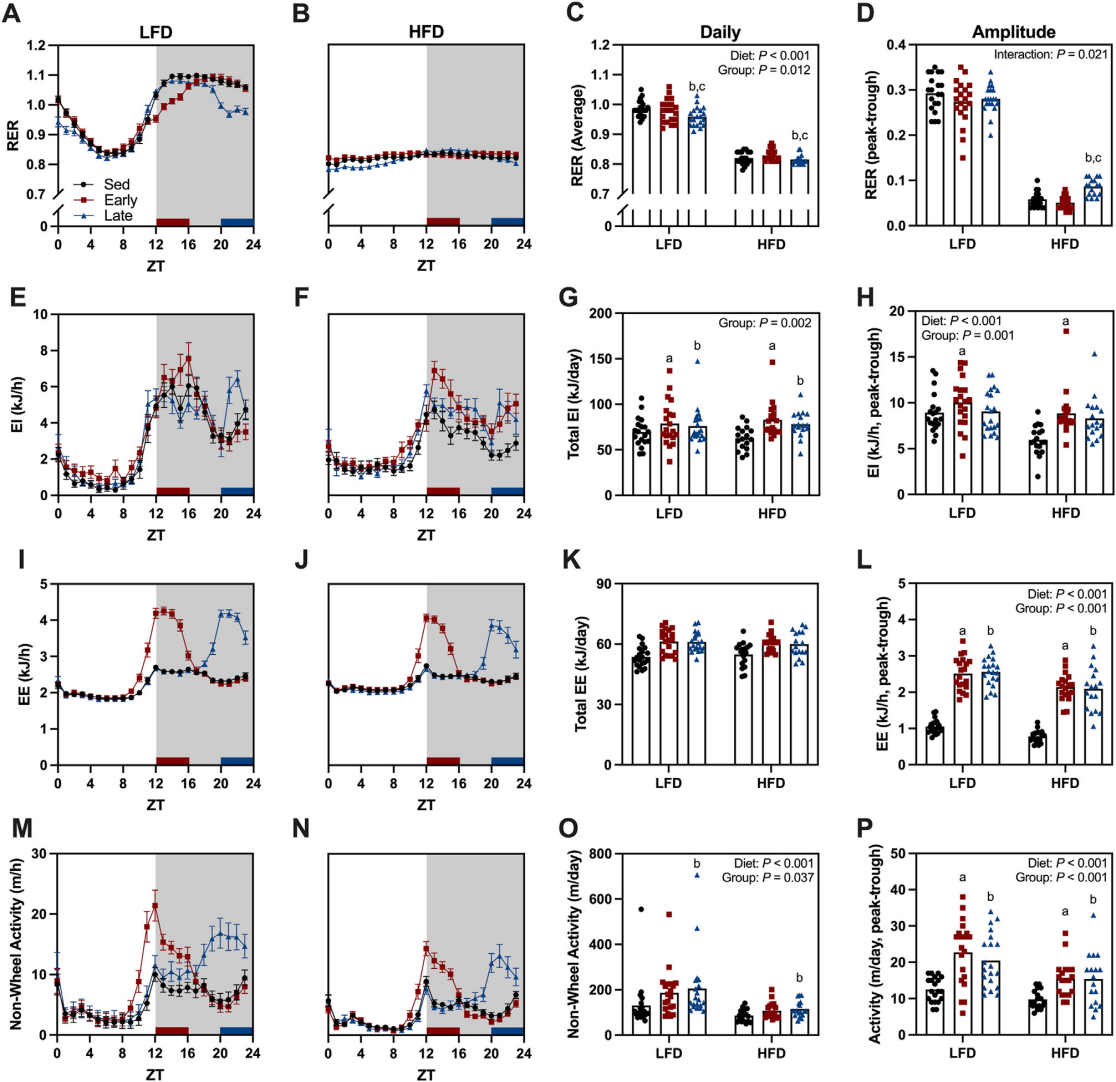
**Altered diurnal metabolism following time-of-day-dependent exercise.** (A–B) Diurnal pattern of the respiratory exchange ratio (RER) in low-fat diet (LFD) and high-fat diet (HFD) mice. (C) Daily average RER and (D) amplitude of daily RER. (E–F) Diurnal pattern in energy intake in LFD and HFD mice. (G) Daily total energy intake and (H) daily energy intake amplitude. (I–J) Diurnal pattern of energy expenditure in LFD and HFD mice. (K) Daily total energy expenditure and (L) daily amplitude in energy expenditure. (M–N) Diurnal pattern in non-wheel activity in LFD and HFD mice. (O) Daily total in non-wheel activity and (P) daily amplitude in non-wheel activity. Data represents averages over a 5-day period taken from week 4 of the wheel running intervention. Data mean ± SE or mean with individual data points representing individual animals (n = 17–20/group). Statistical analysis was performed using a two-way ANOVA. ^a^Sed v Early, ^b^Sed v Late and ^c^Early v Late (*P* < 0.05).

In LFD mice, RER fluctuated substantially throughout the day, but this diurnal variation disappeared entirely in HFD mice (Figure 3A–B). As a result, HFD mice exhibited a lower daily average RER than LFD mice (Figure 3C). Additionally, late wheel access mice had a lower daily average RER compared to both sedentary and early wheel access mice (Figure 3C). This likely stems from the sustained reduction in RER during the light phase, which occurred only in late wheel access mice (Figure 3A–B). Furthermore, HFD late wheel access mice displayed a greater RER amplitude than HFD sedentary and early wheel access mice (Figure 3D).

The wheel running intervention altered the diurnal pattern in energy intake. Early wheel access mice consumed the most energy during the early dark phase, coinciding with the respective wheel access period, whereas late wheel access mice ate more consistently throughout the dark phase (Figure 3E–F). Both early and late wheel access mice consumed more total daily energy than sedentary mice, though no differences existed between the early and late wheel access groups (Figure 3G). Early wheel access mice exhibited a higher energy intake amplitude than sedentary mice, but there were no differences between sedentary and late wheel access mice or between early and late wheel access mice (Figure 3H). Additionally, HFD mice had a lower energy intake amplitude than LFD mice (Figure 3H).

As expected, energy expenditure increased substantially during the early and late wheel access periods. However, these effects were transient and did not alter the overall diurnal energy expenditure pattern (Figure 3I–J). Both early and late wheel access mice expended more total daily energy expenditure than sedentary mice, but no differences were observed between the early and late wheel access groups (Figure 3K). The increased energy expenditure in early and late wheel access mice led to a greater energy expenditure amplitude compared to sedentary mice (Figure 3L), though this amplitude was lower in HFD versus LFD mice (Figure 3L).

HFD-fed mice exhibited lower non-wheel activity levels compared to LFD-fed mice (Figure 3O). The wheel-running intervention also modified non-wheel activity patterns with both early and late wheel access mice displaying peaks in non-wheel running activity that coincided with the respective wheel access window (Figure 3M−N). Late wheel access mice engaged in more total daily non-wheel activity than sedentary mice (Figure 3O), while both early and late wheel access mice exhibited a greater amplitude in non-wheel activity compared to sedentary mice (Figure 3P).

### 3.4 Enhanced insulin sensitivity following late dark phase wheel running

**Figure 4:**
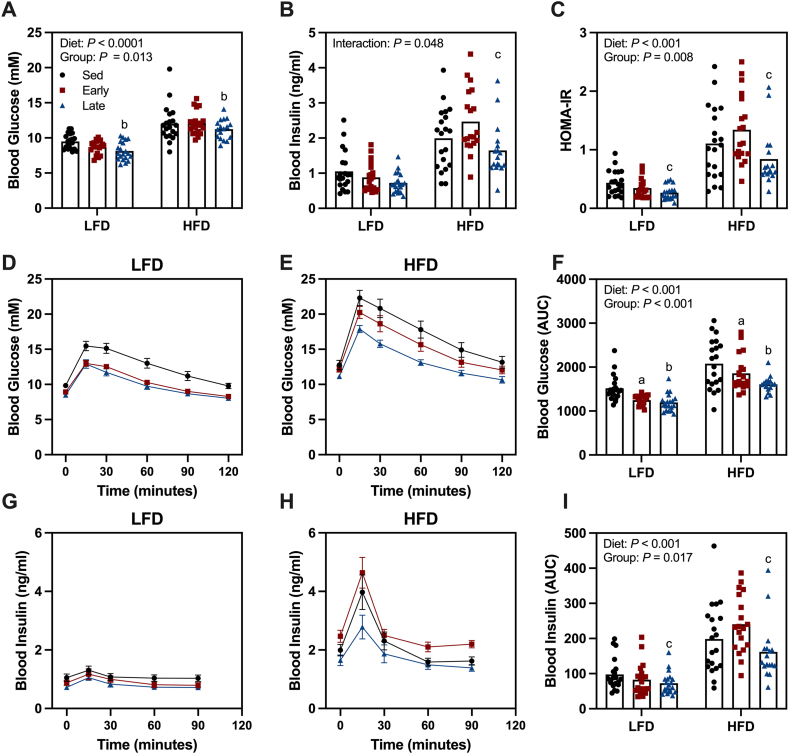
**Enhanced insulin sensitivity following late wheel phase wheel running.** (A) Blood glucose and (B) blood insulin following a 4 h fast. (C) Homeostatic Model Assessment for Insulin Resistance (HOMA-IR) calculated from basal blood glucose and insulin values. (D–E) Assessment of glucose during an oral glucose tolerance test (GTT) in low-fat diet (LFD) and high-fat diet (HFD) mice. (F) Area under the curve (AUC) for blood glucose during the GTT. (G–H) Assessment of blood insulin during the GTT in LFD and HFD mice. (I) AUC for blood insulin during the GTT. Data are reported as mean ± SE or mean values with individual data points representing individual animals (n = 17–20/group). Statistical analysis was performed using a two-way ANOVA. ^a^Sed v Early, ^b^Sed v Late and ^c^Early v Late (*P* < 0.05).

Following the exercise intervention, glucose tolerance was assessed by means of an OGTT. HFD mice exhibited higher basal blood glucose and insulin levels, leading to a higher HOMA-IR compared to LFD mice (Figure 4A–C). Late wheel access mice had lower basal blood glucose levels than sedentary mice, while early wheel access mice showed no differences from sedentary or late wheel access mice (Figure 4A). Basal insulin levels followed a similar pattern, with HFD late wheel access mice showing lower basal insulin levels than HFD sedentary mice (Figure 4B). The lower glucose and insulin levels in late wheel access mice resulted in a lower HOMA-IR compared to early wheel access mice (Figure 4C). HFD feeding impaired glucose tolerance compared to LFD mice (Figure 4F). However, both early and late wheel access mice showed improved glucose tolerance, as indicated by a lower AUC compared to sedentary mice (Figure 4F). During the GTT, HFD-fed mice exhibited higher insulin levels than LFD mice (Figure 4I). Additionally, late wheel access mice had lower insulin AUC during the GTT compared to early wheel access mice (Figure 4I).

### 3.5 Lower tissue lipid deposition following early and late wheel running

**Figure 5:**
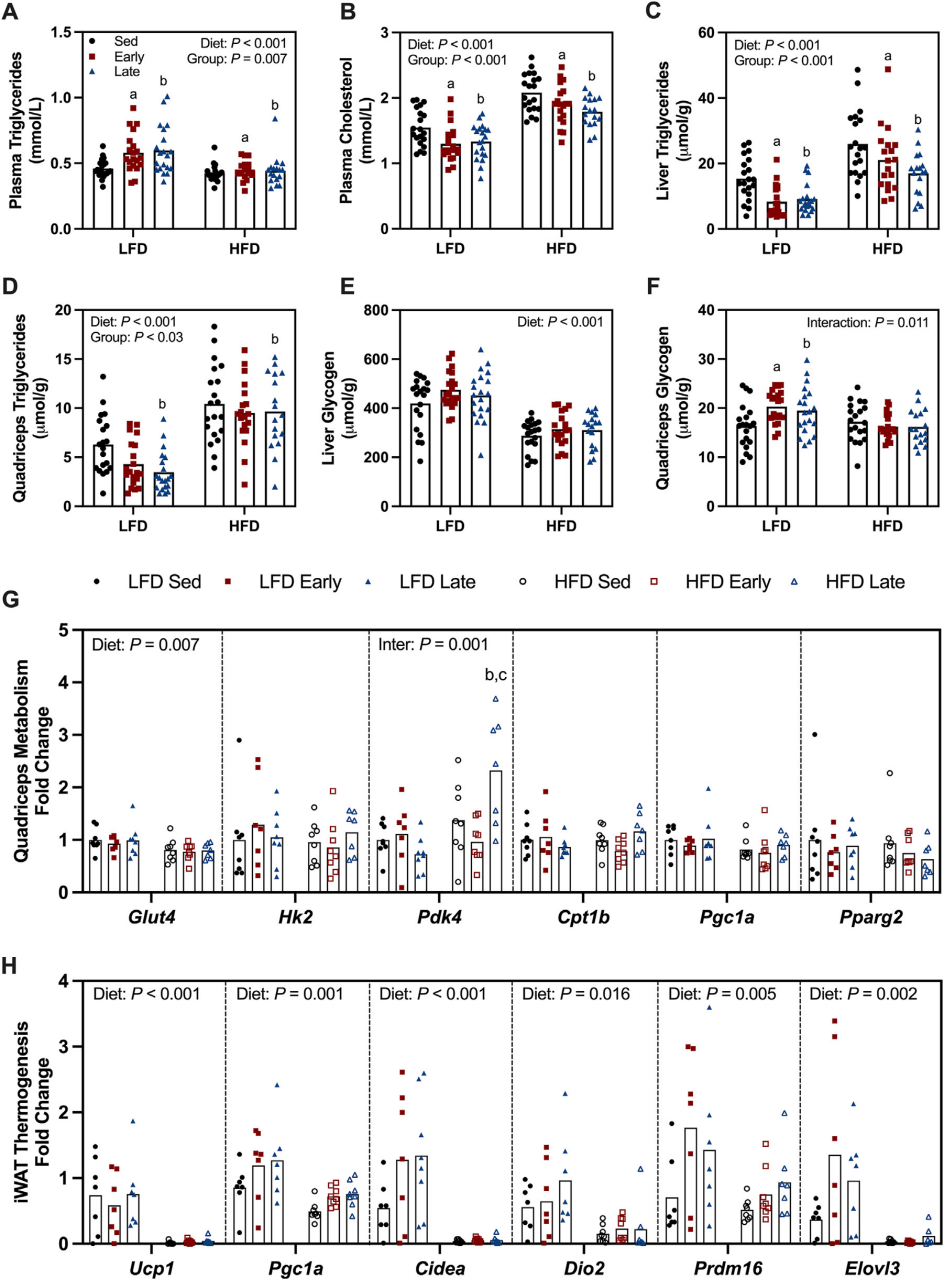
**Reduced ectopic lipid deposition following wheel running intervention.** Plasma triglycerides (A) and cholesterol (B) levels assessed following the wheel running intervention. Liver (C) and quadriceps (D) triglyceride content. Liver (E) and quadriceps (F) glycogen levels. Quadriceps gene expression of glucose transporter 4 (*Glut4*), hexokinase 2 (*Hk2*), pyruvate dehydrogenase kinase 4 (*Pdk4*), carnitine palmitoyltransferase 1B (Cpt1b), peroxisome proliferator-activated receptor gamma coactivator 1-alpha (*Pgc1a*) and peroxisome proliferator-activated receptor gamma (*Pparg2*) (G). Inguinal white adipose tissue (iWAT) gene expression of uncoupling protein 1 (*Ucp1*), peroxisome proliferator-activated receptor gamma coactivator 1-alpha (*Pgc1a*), cell death inducing DFFA like effector a (*Cidea*), iodothyronine deiodinase 2 (*Dio2*), PR/SET domain 16 (*Prdm16*) and ELOVL fatty acid elongase 3 (*Elovl3*) (H). Data are reported as mean ± SE or mean values with individual data points representing individual animals (A–F: n = 17–20/group, G–H: n = 7–8/group). Statistical analysis was performed using a two-way ANOVA. ^a^Sed v Early, ^b^Sed v Late and ^c^Early v Late (*P* < 0.05).

HFD-fed mice showed lower plasma triglyceride levels than LFD mice (Figure 5A). Both early and late wheel access mice exhibited higher plasma triglycerides than sedentary mice, but no differences emerged between early and late wheel access groups (Figure 5A). Similarly, HFD feeding increased plasma cholesterol levels compared to LFD mice (Figure 5B). However, both early and late wheel access mice had lower plasma cholesterol levels than sedentary mice, with no differences between the two exercise groups (Figure 5B).

HFD-fed mice accumulated more triglycerides in liver and skeletal muscle than LFD mice (Figure 5C–D). Both early and late wheel access mice had lower liver triglyceride levels than sedentary mice, but levels did not differ from each other (Figure 5C). Late wheel access mice showed lower quadriceps muscle triglyceride levels than sedentary mice, whereas early wheel access mice displayed no differences compared to sedentary or late wheel access mice (Figure 5D). HFD-fed mice stored less glycogen in the liver than LFD mice, with no differences between wheel access groups (Figure 5E). Conversely, both early and late wheel access mice on LFD diet accumulated more glycogen in quadriceps than sedentary mice (Figure 5F).

### 3.6 Enhanced *Pdk4* expression following late dark phase exercise in HFD

We evaluated the expression of genes commonly associated with skeletal muscle exercise adaptation and adipose tissue thermogenesis in quadriceps and iWAT, respectively. After dietary and exercise interventions we observed no changes in the expression of hexokinase 2 (*Hk2*), carnitine palmitoyltransferase 1B (*Cpt1b*), peroxisome proliferator-activated receptor gamma coactivator 1-alpha (*Pgc1a*) and peroxisome proliferator-activated receptor gamma (*Pparg2*) (Figure 5G). However, the dietary intervention reduced glucose transporter 4 (*Glut4*) expression in quadriceps of HFD mice compared to LFD mice (Figure 5G). Additionally, we observed an interaction between exercise and diet in pyruvate dehydrogenase kinase 4 (*Pdk4*) expression. Specifically, *Pdk4* expression increased in the quadriceps of late wheel access mice compared to both sedentary and early wheel access mice, but only within the HFD group (Figure 5G).

In iWAT, genes related to thermogenesis, including uncoupling protein 1 (*Ucp1*), *Pgc1a*, cell death inducing DFFA like effector a (*Cidea*), iodothyronine deiodinase 2 (*Dio2*), PR/SET domain 16 (*Prdm16*) and ELOVL fatty acid elongase 3 (*Elovl3*), showed reduced expression in HFD mice compared to LFD mice. However, exercise did not affect the expression of thermogenesis-related genes in iWAT (Figure 5H).

### 3.7 Diet-induced alteration of the core clock within peripheral tissues

**Figure 6:**
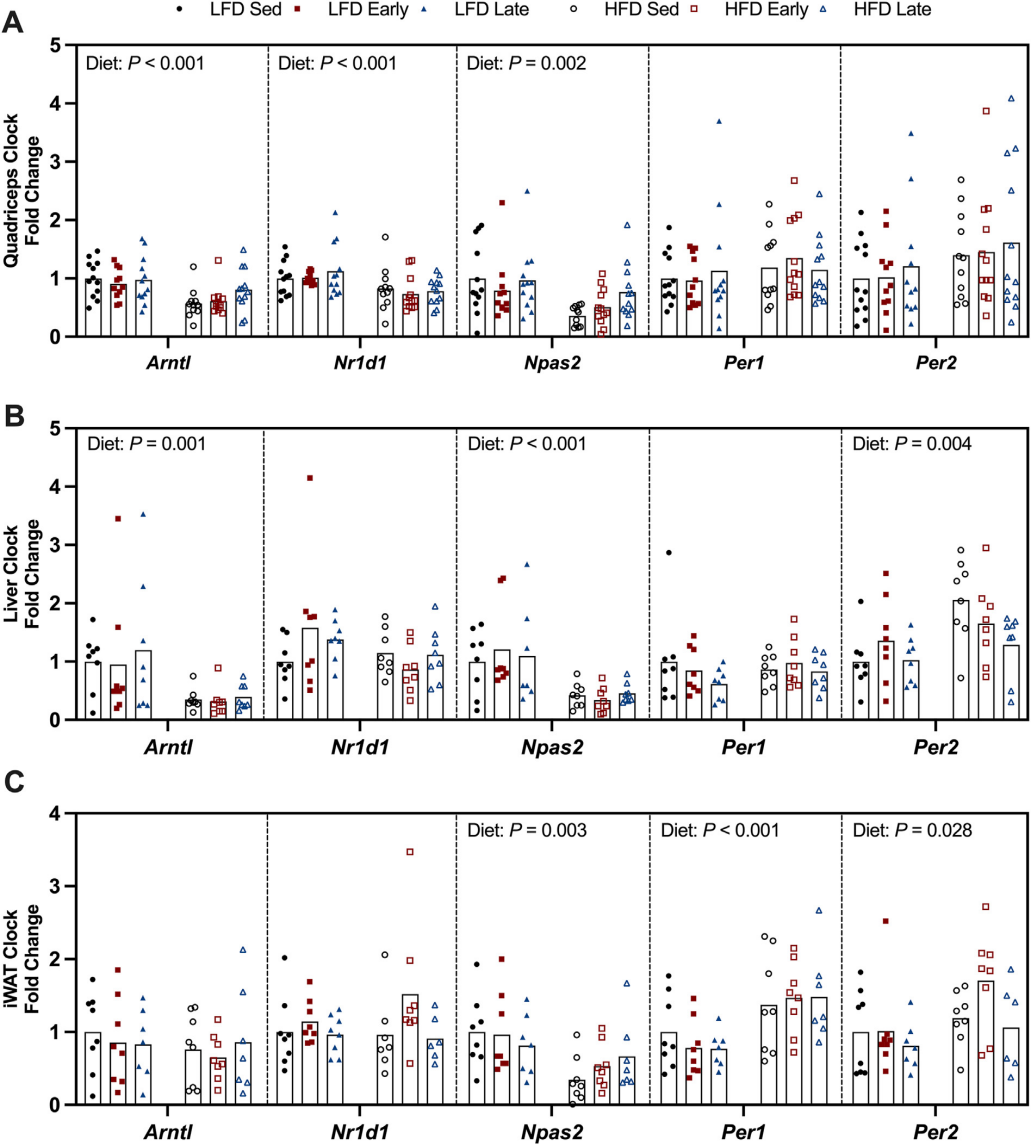
**HFD-induced alterations in tissue-specific clocks.** Expression of core clock-associated genes, including basic helix-loop-helix ARNT like 1 (*Arntl*), nuclear receptor subfamily 1 group D member 1 (*Nr1d1*), neuronal PAS domain protein 2 (*Npas2*), period 1 (*Per1*) and period 2 (*Per2*) in quadriceps (A), liver (B) and inguinal white adipose tissue (iWAT) (C). Data are reported as mean values with individual data points representing individual animals (n = 7–12/group). Statistical analysis was performed using a two-way ANOVA. ^a^Sed v Early, ^b^Sed v Late and ^c^Early v Late (*P* < 0.05).

We then investigated the tissue specific expression of core clock-related genes. Diet, rather than exercise, primarily influenced core clock gene expression, with tissue-specific variations. For example, HFD feeding reduced nuclear receptor subfamily 1 group D member 1 (*Nr1d1*) expression in quadriceps of HFD-fed mice, while increasing period 1 (*Per1*) expression increased in iWAT (Figure 6A,C). In both liver and quadriceps, HFD feeding decreased basic helix-loop-helix ARNT like 1 (*Arntl*) expression compared to LFD-fed mice (Figure 6A–B), though *Arntl* expression remained unchanged in iWAT (Figure 6C). HFD feeding also increased period 2 (*Per2*) expression in liver and iWAT but did not alter expression in quadriceps (Figure 6A–C). Among all the analyzed tissues, only neuronal PAS domain protein 2 (*Npas2*) expression was consistently affected by diet (Figure 6A–C). Specifically, HFD feeding reduced *Npas2* expression in liver, quadriceps and iWAT of HFD-fed mice compared to LFD-fed mice (Figure 6A–C).

## 4 Discussion

Utilizing a model in which HFD-fed mice are provided VWR access in either the early (ZT12-16) or late (ZT20-0) dark phase, we investigated the time-of-day-dependent effects of exercise on energy homeostasis. Our findings demonstrate that exercise interventions, irrespective of timing, confer metabolic health benefits, including attenuated body weight gain, improved glucose tolerance, and reduced ectopic lipid deposition. However, certain metabolic adaptations were specific to late dark phase exercise, including a greater attenuation of body weight gain, enhanced fat oxidation during the light phase, and improved insulin sensitivity.

The time-of-day-dependent effects of exercise on metabolic health include a reduction in body weight gain following exercise conducted during the late dark phase [[24], [25], [26]]. Consistent with this, we observed a greater attenuation of body weight gain in the late dark phase exercise group, primarily driven by a reduction in fat mass. This shift resulted in a more metabolically favorable body composition, with a higher proportion of lean mass relative to body weight. The reduction in body weight gain is likely attributed to alterations in energy balance. To investigate this further, mice were housed in metabolic cages throughout the intervention, allowing for continuous assessment of energy homeostasis.

Both early and late dark phase exercise increased energy expenditure, driven by VWR and elevated non-wheel activity compared to sedentary mice. However, total energy intake remained unchanged across groups, suggesting that weight gain attenuation was primarily mediated by increased energy expenditure. Notably, this does not explain why the late dark phase exercise group exhibited a greater reduction in weight gain than the early dark phase group. Our data align with previous findings showing no significant time-of-day-dependent differences in exercise-induced energy expenditure [26]. Additionally, no differences were observed in running speed, daily distance covered, total training volume, or non-wheel activity between early and late dark phase exercise groups, suggesting that increased energy expenditure occurs independently of exercise timing. Tissue-specific metabolic adaptations may also play a role, as exercise promotes the browning of white adipose tissue in rodents [30]. However, exercise did not alter the expression of genes commonly associated with adipose thermogenesis (*Ucp1*, *Pgc1a*, *Cidea*, *Dio2*, *Prdm16*, *Elovl3*).

Reductions in body weight gain may result from decreased energy intake however, total energy intake appears to be unaltered by time-of-day-dependent exercise in mouse models [24,26]. Using metabolic chambers, we monitored every feeding event and quantified total energy intake throughout the exercise intervention. Cumulative energy intake and average energy intake per feeding event were similar between the early and late wheel access groups. However, the diurnal pattern of energy intake differed. For example, early wheel access mice exhibit a greater peak in energy intake during the early dark phase, whereas late wheel access mice fed more consistently throughout the dark phase. Alterations in diurnal feeding patterns, such as time-restricted or light-phase feeding, influence body composition in rodents fed a HFD [31,32]. Furthermore, the reduction in body weight gain following late dark phase exercise has been suggested to be driven by the pattern of food intake surrounding the exercise bouts [26]. Specifically, when exercise was preceded by food intake, HFD-induced weight gain was attenuated compared to when food intake was restricted to the post-exercise period [26].

While total energy intake remains similar between early and late wheel access groups, differences in energy absorption may contribute to variations in body weight gain. Notably, both time-restricted feeding [33] and exercise [25] interventions alter the gut microbiota composition. Additionally, late dark phase exercise increases the abundance of gut microbiota that produce short-chain fatty acids, which influence metabolism in peripheral tissues such as adipose tissue [25,34]. Therefore, future studies should consider the measurements of assimilated calories alongside food intake to obtain a more accurate picture of energy intake and absorption [35].

The diurnal pattern of substrate metabolism is altered following time-of-day-dependent exercise interventions [26]. Specifically, we confirm that late dark phase running reduces RER during the subsequent light phase [26]. Notably, we observed a greater amplitude in RER within the HFD-fed group following late dark phase exercise, suggesting an enhancement in metabolic flexibility. This finding is further supported by the increased expression of *Pdk4* in skeletal muscle, specifically during the late dark phase in HFD-fed mice. PDK4 inhibits the pyruvate dehydrogenase complex (PDC), thereby promoting fatty acid oxidation [36]. Collectively, these results provide evidence for enhanced fat oxidation following late dark phase exercise, potentially contributing to improved body composition. This effect may be linked to the energy status surrounding the exercise period. For instance, in both LFD- and HFD-fed mice, liver glycogen levels reach a nadir at the onset of the dark phase (ZT12) [27]. Therefore, exercise in the early dark phase may be interpreted as a train-low paradigm (training with low glycogen levels). Both during exercise and within the post-exercise period, early dark phase exercise coincides with a period of elevated food intake to replenish energy stores. Conversely, late dark phase exercise is preceded by elevated food intake and glycogen stores should be elevated at onset of exercise. Following late dark phase exercise, mice enter the inactive phase and food intake is reduced, which may promote a sustained period of fat oxidation following late dark phase exercise.

We also observed an enhanced anticipatory response at the onset of the dark phase in the early dark phase exercise group, potentially indicating a divergent stress response between exercise conditions. Voluntary wheel running is known to elevate corticosterone levels at the onset of the dark phase [37] and alterations in stress hormones may influence diurnal patterns of substrate metabolism. Therefore, future studies should consider the measurement of stress hormones across the diurnal cycle.

Exercise training improves peripheral insulin sensitivity in rodents and humans [38,39]; however, the effects of time-of-day-dependent exercise on whole-body glucose tolerance are unknown. Following 4 weeks of time-restricted VWR, glucose tolerance improved in early and late dark phase running groups compared to sedentary mice. The exercise-induced improvements in glucose tolerance were associated with lower insulin secretion during the GTT, particularly following late dark phase exercise. Furthermore, reduced fasting glucose and insulin levels, along with lower HOMA values in late dark phase exercise groups, indicate enhanced insulin sensitivity. However, a prior report indicates that insulin-stimulated glucose uptake in skeletal muscle and adipose tissue is unaltered following early and late dark phase exercise [24]. Since we did not directly assess tissue-specific insulin sensitivity, determining whether the improvements in glucose tolerance were due to enhanced peripheral insulin sensitivity or other mechanisms remain unclear.

Discrepancies in the effects of exercise timing on metabolic outcomes may be related to the C57BL/6 (NTac v BomTac) sub-strains utilized between studies, which display notable metabolic differences following HFD-feeding [40,41]. Conversely, the overall experimental design including the length of dietary and exercise intervention were similar [24]. Therefore, assuming the current intervention did not improve skeletal muscle insulin sensitivity, the enhanced glucose tolerance may be related to hepatic glucose production. Wheel running interventions improve hepatic insulin sensitivity [42]; however, whether the effects are time-of-day-dependent is currently unclear. In humans, afternoon exercise improves insulin sensitivity and basal hepatic glucose production in metabolically compromised individuals [43,44]. Therefore, further investigations examining the time-of-day-dependent effects of exercise on hepatic metabolism may be warranted.

Exercise training influences the skeletal muscle clock and increases the abundance of core clock associated proteins [3,24]. Despite this, we observed no exercise-induced effects on core-clock associated gene expression (*Arntl*, *Nr1d1*, *Npas2*, *Per1*, *Per2*) within skeletal muscle, liver or iWAT. Furthermore, the exercise intervention did not alter the expression levels of several genes commonly associated with exercise adaptation in skeletal muscle (*Hk2*, *Glut4*, *Cpt1b*, *Pgc1a* and *Pparg2*). Given that tissue samples were collected ∼72 h following the last exercise bout, this finding is perhaps unsurprising. To appropriately assess the molecular core clock, serial tissue sampling across the full circadian cycle is required. In addition, the assessment of protein abundance or enzymatic activity may uncover the molecular adaptations underpinning the whole-body metabolic effects observed.

In conclusion, VWR interventions in a model of diet-induced obesity confers multiple metabolic adaptations irrespective of time-of-day, including reduced body weight gain, enhanced glucose tolerance, and reduced ectopic lipid deposition. However, a greater attenuation in body weight gain, also with enhancements in metabolic flexibility and insulin sensitivity, was observed exclusively following late dark phase exercise. Divergent adaptations in body composition are likely driven by alterations in the diurnal pattern of energy intake and substrate metabolism. Overall, our data supports the use of exercise interventions as a therapeutic tool to combat multiple facets of metabolic disease regardless of the time of day. However, the enhanced metabolic benefits observed with late dark phase exercise suggest that optimizing exercise timing or the metabolic state in which exercise is performed could further enhance its efficacy.

## CRediT authorship contribution statement

**Stephen P. Ashcroft:** Writing – review & editing, Writing – original draft, Methodology, Investigation, Formal analysis, Data curation, Conceptualization. **Amy M. Ehrlich:** Writing – review & editing, Writing – original draft, Methodology, Investigation, Formal analysis, Data curation, Conceptualization. **Krzysztof Burek:** Writing – review & editing, Methodology, Formal analysis. **Logan A. Pendergrast:** Writing – review & editing, Investigation, Formal analysis. **Caio Y. Yonamine:** Writing – review & editing, Investigation, Formal analysis. **Jonas T. Treebak:** Writing – review & editing, Writing – original draft, Supervision, Resources, Project administration, Conceptualization. **Juleen R. Zierath:** Writing – review & editing, Writing – original draft, Resources, Project administration, Funding acquisition, Conceptualization.

## Duality of interest

No potential conflicts of interest relevant to this article were reported.

## Funding sources

The Novo Nordisk Foundation Center for Basic Metabolic Research is an independent research center at the Faculty of Health and Medical Sciences, University of Copenhagen, Denmark, partially funded by an unrestricted donation from the Novo Nordisk Foundation (NNF18CC0034900, NNF23SA0084103). This research was supported by a Novo Nordisk Foundation Challenge Grant (NNF14OC0011493) and the Swedish Research Council (2015-00165) the European Research Council (ERC-2023-AdG 101142093), a Wallenberg Scholar grant from Knut and Alice Wallenberg Foundation (2023.0312), to J.R.Z.

## Declaration of competing interest

The authors declare the following financial interests/personal relationships which may be considered as potential competing interests:

Juleen R. Zierath reports financial support was provided by Swedish Research Council. Juleen R. Zierath reports financial support was provided by Swedish Diabetes Foundation. Juleen R. Zierath reports financial support was provided by Diabetes Wellness Foundation Network Sweden. Juleen R. Zierath reports financial support was provided by Novo Nordisk Foundation. Juleen R. Zierath reports financial support was provided by Swedish Research Council for Sport Science. Juleen R. Zierath reports financial support was provided by Swedish Foundation for Strategic Research. If there are other authors, they declare that they have no known competing financial interests or personal relationships that could have appeared to influence the work reported in this paper.

## Data Availability

Data will be made available on request.
